# Arthropod venom Hyaluronidases: biochemical properties and potential applications in medicine and biotechnology

**DOI:** 10.1186/s40409-015-0042-7

**Published:** 2015-10-22

**Authors:** Karla C F Bordon, Gisele A. Wiezel, Fernanda G. Amorim, Eliane C. Arantes

**Affiliations:** Department of Physics and Chemistry, School of Pharmaceutical Sciences of Ribeirão Preto, University of São Paulo (USP), Avenida do Café, s/n, Ribeirão Preto, SP 14.040-903 Brazil

**Keywords:** Hyaluronidase, Scorpion, Spider, Caterpillar, Hymenoptera, Insects, Cloning, Heterologous expression, PEGylation, Biotechnological applications

## Abstract

Hyaluronidases are enzymes that mainly degrade hyaluronan, the major glycosaminoglycan of the interstitial matrix. They are involved in several pathological and physiological activities including fertilization, wound healing, embryogenesis, angiogenesis, diffusion of toxins and drugs, metastasis, pneumonia, sepsis, bacteremia, meningitis, inflammation and allergy, among others. Hyaluronidases are widely distributed in nature and the enzymes from mammalian spermatozoa, lysosomes and animal venoms belong to the subclass EC 3.2.1.35. To date, only five three-dimensional structures for arthropod venom hyaluronidases (*Apis mellifera* and *Vespula vulgaris*) were determined. Additionally, there are four molecular models for hyaluronidases from *Mesobuthus martensii*, *Polybia paulista* and *Tityus serrulatus* venoms. These enzymes are employed as adjuvants to increase the absorption and dispersion of other drugs and have been used in various off-label clinical conditions to reduce tissue edema. Moreover, a PEGylated form of a recombinant human hyaluronidase is currently under clinical trials for the treatment of metastatic pancreatic cancer. This review focuses on the arthropod venom hyaluronidases and provides an overview of their biochemical properties, role in the envenoming, structure/activity relationship, and potential medical and biotechnological applications.

## Introduction

**Fig. 1:**
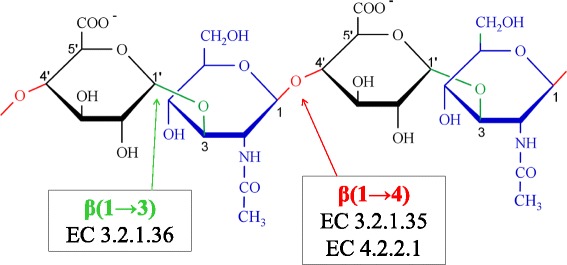
Structure of hyaluronan. The repeating disaccharide units of *N*-acetyl-β-D-glucosamine (GlcNAc) and β-D-glucuronic acid (GlcUA) linked via alternating β-1,3 (highlighted in green) and β-1,4 glycosidic bonds (highlighted in red) are shown. The hyaluronidases EC 3.2.1.36 cleave the β-1,3 glycosidic bond, EC 3.2.1.35 the β-1,4 glycosidic bond and the EC 4.2.2.1 the β-1,4 glycosidic bond by elimination, yielding a double bond between carbons 4’ and 5’

Hyaluronidases are glycosidases that cleave preferentially the hyaluronan in the extracellular matrix (ECM) found in soft connective tissues. Hyaluronan is a linear polysaccharide formed by repeating disaccharide units of *N*-acetyl-β-D-glucosamine (GlcNAc) and β-D-glucuronic acid (GlcUA) linked via alternating β-1,3 and β-1,4 glycosidic bonds (Fig. 1). It acts as an impact absorber and lubricant in the articulations, playing a relevant structural role in maintaining the architecture of the ECM. This is rendered possible since hyaluronan interacts with many water molecules, assuming great viscoelasticity [1–3].

Hyaluronidases increase up to 20 times the infusion rates and penetration of molecules up to 200 nm in diameter because of the cleavage of hyaluronan, reducing the obstacle that the interstitial matrix presents to fluid and drug transfer [4].

The hyaluronidase activity was identified for the first time by Duran-Reynals in 1928, but the term hyaluronidase was introduced only in 1940 [5, 6]. These enzymes are widely distributed in nature and have been reported in animal venoms (such as snake [7, 8], wasp [9], scorpion [10, 11], bee [12], hornet [13], freshwater stingray [14], fish [15], spider [16], lizard [17] and caterpillar [18, 19] venoms), human organs (testis, eye, skin, spleen, liver, kidneys, uterus) and corporal fluids (placenta, tears, blood, sperm) [20, 21], bacteria [22], hookworm [23], fungi [24], bacteriophages [25], crustaceans [26], mollusks [27], leeches [28], other animal tissues [29, 30] and malignant tumors [31]. The first hyaluronidase was isolated from bovine testis [29] and has been legally sold in the USA since 1948 [32, 33]. However, the first venom hyaluronidase was isolated only in 1973 from *Dugesiella hentzi* tarantula venom [34]. Usually, hyaluronidases are present in venoms in such low proportion that they are not detectable through proteomic analyses [35].

**Fig. 2:**
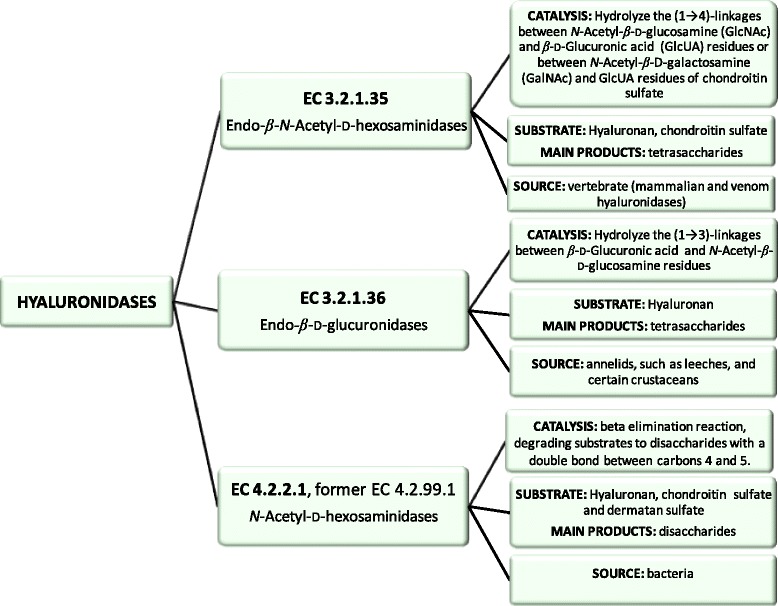
The three major groups of hyaluronidases. The EC numbers, catalysis, substrates, main products and sources of each hyaluronidase group are shown

Hyaluronidases are classified into three major groups [21, 36, 37]. They degrade preferentially hyaluronan, though different reaction mechanisms are involved (Fig. 2). The first group (EC 3.2.1.35) includes vertebrate enzymes (e. g. mammalian and venom hyaluronidases) that are endo-β-*N*-acetyl-D-hexosaminidases and hydrolyze the β-1,4 glycosidic bond between GlcNAc and GlcUA residues in hyaluronan to the tetrasaccharide (GlcUA-GlcNAc-GlcUA-GlcNAc) as the main product. These enzymes are also able to cleave chondroitin sulfate. The second group (EC 3.2.1.36) is composed of hyaluronidases from annelids, such as leeches and certain crustaceans. These enzymes are endo-β-D-glucuronidases that degrade hyaluronan to the tetrasaccharide (GlcNAc-GlcUA-GlcNAc-GlcUA) by hydrolyzing the β-1,3 glycosidic bond between GlcUA and GlcNAc residues in hyaluronan. The third one (EC 4.2.2.1, former EC 4.2.99.1) is represented by bacterial *N*-acetyl-D-hexosaminidases that cleave the β-1,4 glycosidic bond by a beta elimination reaction, degrading hyaluronan, chondroitin sulfate and dermatan sulfate to disaccharides with a double bond between carbons 4 and 5.

The hyaluronidase activity is modulated by various activators (adrenalin, histamine and acid phosphatase found in prostate, liver, kidney, erythrocytes and platelets) and inhibitors (antihistamines, salicylates, heparin, dicoumarin, vitamin C and flavonoids) [38, 39].

This enzyme has been used as an adjuvant to increase the absorption and dispersion of injected drugs [32, 40], to reduce edema [41, 42] and local side effects in tissues [32], and as a healing-promoting agent for skin lesions [43]. In 2005, a highly purified recombinant human hyaluronidase (rHuPH20) was approved by the FDA [32, 44]. A phase IV clinical trial using this enzyme associated to insulin analogs is under study for the treatment of type 1 diabetes [45, 46]. Additionally, a biopharmaceutical product containing rHuPH20 was approved for the treatment of adult patients with primary immunodeficiency in 2014 [40], and another one containing a PEGylated form of rHuPH20 (PEGPH20) has been under a phase II clinical trial for the first-line treatment of metastatic pancreatic cancer [47].

Many hyaluronidases (from prokaryotes and eukaryotes) have been studied and a great diversity in their activity can be observed among different species. Such diversity has been demonstrated by the optimal pH, isoelectric point, number of isoforms, molecular mass, substrate specificity and sensitivity in the presence of various modulators [48].

Hyaluronidases are usually classified as acid-active (maximum activity from pH 3 to pH 4) or neutral-active enzymes (maximum activity from pH 5 to pH 6) [49]. Hyaluronidases isolated from snake, bee and scorpion venoms are active in pH from 4 to 6 and present a molecular mass between 33 and 100 kDa [50–52]. Cevallos et al. [50] observed that venom hyaluronidases from some invertebrates (*Dolichovespula maculata*, *Vespula germanica*, *Pogonomyrmex rugosus* and *Centruroides limpidus limpidus*) presented less than 50 kDa, while those from vertebrates (bovine, *Heloderma horridum horridum*, *H. suspectum suspectum*, *Lachesis muta*, *Crotalus basiliscus*, *Bothrops asper* and *Micrurus nigrocinctus*) are comprised of hyaluronidases larger than 60 kDa and more than one active isoform may be present. On the other hand, small hyaluronidases (lower than 60 kDa) have already been identified in vertebrate venoms [7] and enzymes presenting more than 50 kDa have already been isolated from invertebrate ones [53].

About two-thirds of all named species in the world, which corresponds to approximately 1,000,000 species, belong to the phylum Arthropoda and the class Insecta represents about 80 % of this phylum. The arthropods have significant economic impact and affect all aspects of the human life. Examples include the pollination of crops and diseases spread by insects and ticks [54]. The present paper reviews the hyaluronidases present in arthropod venoms as well as their potential applications in medicine and biotechnology.

## Review

### Role of arthropod venom hyaluronidases in envenoming

Hyaluronidases are not toxic by themselves, but they potentiate the effect of other toxins present in venoms, contributing to the local and systemic effects of envenoming [16, 55]. Furthermore, they are described as allergens from arthropod venoms, being able to induce severe and fatal anaphylactic IgE-mediated reactions in humans [13, 56]. These enzymes are known as “spreading factors”, a concept firstly introduced by Duran-Reynals in 1933 [11, 57]. This action was experimentally confirmed [17], resulting in the hydrolysis of hyaluronan and chondroitin sulfates A and C, which promotes the diffusion of toxins through the tissues and blood circulation of the victim/prey [7, 8, 17, 58, 59].

The hyaluronidase plays a key role in the Pararama associated phalangeal periarthritis observed after the envenoming caused by the caterpillar *Premolis semirufa* [60]. The enzyme from the spider *Hippasa partita* indirectly potentiated the myotoxicity of VRV-PL-VIII myotoxin and the effect of hemorrhagic complex-I [16]. Similar results were observed with the recombinant hyaluronidase from the spider *Loxosceles intermedia*, which increased the effect of the recombinant dermonecrotic toxin LiRecDT1 [55]. The enzyme from telmophage insects is responsible for extending the feeding lesion and diffusing anti-hemostatic agents into the host tissue [61].

Additionally, the hyaluronidase from *Tityus serrulatus* scorpion venom potentiates the activity of Ts1, the major neurotoxin present in this venom, increasing the serum levels of creatine kinase (CK), lactate dehydrogenase (LD) and aspartate aminotransferase (AST) [10]. Therefore, to assess the importance of hyaluronidase in the scorpion envenoming process, the toxic effects of *T. serrultatus* venom were evaluated after the *in vitro* and *in vivo* inhibition and immunoneutralization of the hyaluronidase activity by anti-hyaluronidase serum produced in rabbits [62]. *In vivo* neutralization assays using anti-hyaluronidase serum inhibited or delayed death of mice. The use of aristolochic acid, a pharmacological inhibitor of hyaluronidase, also inhibited death. On the other hand, the survival of mice was reversed after the addition of native hyaluronidase to pre-neutralized venom, showing that hyaluronidase plays a critical role in systemic envenoming [62]. Therefore, inhibitors of the hyaluronidase activity are potential first aid agents to treat envenoming cases [62, 63].

### Structure of hyaluronidases

There are 128 and 92 known primary sequences deposited in the NCBI and UniProt databanks, respectively, for hyaluronidases belonging to 53 genera divided into the classes Arachnida, Chilopoda and Insecta from the phylum Arthropoda (Table 1). All deposited sequences were evidenced at transcript level, with the exception of those from *Phoneutria*, *Tityus* and *Dolichovespula*, which were evidenced at protein level.

**Table 1:** Hyaluronidases from the phylum Arthropoda

Class	Order	Members	Family	Genus	Number of entries (NCBI; UniProt)	Ref.
Arachnida	Araneae	Spiders	Ctenidae	*Phoneutria*	1; 1 - F^a^	—
			Sicariidae	*Loxosceles*	2; 1	[55]
			Theraphosidae	*Brachypelma*	1; 1 - F	[82]
	Scorpiones	Scorpions	Bothriuridae	*Cercophonius*	1; 1 - F	[129]
			Buthidae	*Hottentotta*	1; 1 - F	[130]
				*Isometroides*	1; 1 - F	[129]
				*Mesobuthus*	3; 1	[76]
				*Tityus*	5; 5 - F*(2)	[62, 74, 131]
			Urodacidae	*Urodacus*	1; 1 - F	[129]
Chilopoda	Geophilomorpha	Centipedes	Linotaeniidae	*Strigamia*	0; 2	—
Insecta	Blattodea	Termites	Rhinotermitidae	*Coptotermes*	1; 1	—
	Coleoptera	Beetles	Curculionidae	*Dendroctonus*	2; 2 - F	[132]
			Tenebrionidae	*Tribolium*	2; 1	[133]
	Diptera	Biting horseflies	Tabanidae	*Tabanus*	1; 1	[134]
		Biting midges	Ceratopogonidae	*Culicoides*	4; 4	[135–138]
		Black flies	Simuliidae	*Simulium*	1; 1	[139]
		Midges	Chaoboridae	*Corethrella*	1; 1	[140]
		Mosquitos	Culicidae	*Aedes*	6; 4	[141]
				*Anopheles*	4; 2	[142, 143]
				*Culex*	9; 5	—
				*Psorophora*	1; 1 - F	[144]
		Moth flies	Psychodidae	*Lutzomyia*	2; 2	—
				*Phlebotomus*	4; 4	[145–147]
	Hemiptera	Aphids	Aphididae	*Acyrthosiphon*	1; 1	—
		Assassin bug	Reduviidae	*Rhodnius*	0; 3 - F	—
				*Triatoma*	1; 1 - F	—
	Hymenoptera	Ants	Formicidae	*Acromyrmex*	1; 1	[148]
				*Atta*	0; 1	[149]
				*Camponotus*	1; 1	[150]
				*Cerapachys*	1; 1	[151]
				*Harpegnathos*	1; 1	[150]
				*Solenopsis*	1; 1 - F	[152]
		Bees	Apidae	*Apis*	13; 3	[64, 118]
				*Bombus*	2; 0	—
			Megachilidae	*Megachile*	1; 0	—
		Parasitoid wasps	Braconidae	*Chelonus*	4; 4 - F	—
				*Glyptapanteles*	5; 5	—
				*Meteorus*	1; 1	—
				*Microplitis*	3; 3	—
		Pteromalid parasitoid wasps	Pteromalidae	*Nasonia*	1; 0	—
		Spider wasps	Pompilidae	*Anoplius*	1; 1	—
		Wasps	Vespidae	*Dolichovespula*	3; 1 - ^a^	[13]
				*Eumenes*	1; 1	[153]
				*Orancistrocerus*	1; 1	[154]
				*Polistes*	2; 1 - F	—
				*Polybia*	2; 2 - F	[9]
				*Rhynchium*	1; 1	—
				*Vespa*	2; 1	[155]
				*Vespula*	12; 6	[65]
	Isoptera	Dampwood termites	Termopsidae	*Zootermopsis*	1; 1 - F	[156]
	Lepidoptera	Butterflies	Nymphalidae	*Danaus*	2; 2	[157]
		Silkmoths	Bombycidae	*Bombyx*	1; 1	[158]
	Phthiraptera	Lice	Pediculidae	*Pediculus*	8; 4	[159]
TOTAL					128; 92	

^a^Evidence at protein level (all the others at transcript level); F: fragment; —: unpublished

The first three-dimensional (3D) structure reported for a hyaluronidase belonging to the family 56 of glycoside hydrolases was reported for the enzyme from *Apis mellifera* venom in 2000 [PDB: 1FCQ; 1FCU; 1FCV] [64]. The overall topology of hyaluronidases from this family resembles a classical (β/α)_n_ triosephosphate isomerase (TIM) barrel, where n is equal to 8 in the hyaluronidase from *A. mellifera* venom and 7 in those from *Vespula vulgaris* [PDB: 2ATM] and *P. paulista* [Pp–Hyal, PMDB: PM0077230] venoms [9, 64, 65].

Snake and human hyaluronidases present five disulfide bonds [8, 66]. The disulfide bonds Cys332–Cys343, Cys336–Cys371 and Cys373–Cys383 are part of the epidermal growth factor-like (EGF-like) domain [62]. The enzymes from *A. mellifera*, *V. vulgaris* and *P. paulista* venoms show two disulfide bonds (Cys17–Cys307 and Cys183–Cys196) [9, 64, 65], which are located in the catalytic domain and well conserved in venom hyaluronidases [62]. On the other hand, the enzymes from *T. serrulatus* venom (TsHyal-1 and TsHyal-2, whose numbers of deposit were not stated) exhibit six disulfide bonds common to all known Arachnida hyaluronidases [62]. The sixth disulfide bond (Cys172–Cys215), found only in the Arachnida hyaluronidases, may reinforce the stability of their catalytic site [62].

On the basis of N-glycosylation, the recombinant hyaluronidase from *L. intermedia* presents four putative N-glycosylation sites in its structure; the enzyme from *A. mellifera* venom shows one of four possible sites [55, 64]. The one from *V. vulgaris* venom has three of five possible sites, the one from *P. paulista* venom shows three putative glycosylation sites, the BmHYI from *Mesobuthus martensii* venom presents five potential N-glycosylation sites (the number of deposit for the molecular model was not stated), while TsHyal-1 and TsHyal-2 from *T. serrulatus* venom has seven and ten putative glycosylation sites, respectively [9, 62, 65, 67].

Besides the fact that N-glycosylation sites are not conserved between TsHyal-1 and TsHyal-2, the isoforms from *T. serrulatus* venom show a variation in the active site groove in position 219. TsHyal-1 has a tyrosine (Y), while TsHyal-2 has a histidine (H) at the same position, which may cause different substrate specificity [62]. A mutation in the positioning residue Y247 in human Hyal-4 (equivalent to Y219 in TsHyal-1) altered the substrate specificity [68]. Among the known primary sequences of hyaluronidase, only TsHyal-2 has a histidine (H) in the position 219 [62].

The residues Ser299, Asp107, and Glu109, located at surface-exposed regions of the Pp-Hyal (*P. paulista* hyaluronidase) structure, on opposite sides of the cavity, interact with the polar hydroxyl nitrogen atoms of hyaluronan and with potential antibody-binding sites (five conformational and seven linear epitopes located at surface-exposed regions of the structure) [9]. These residues are of great importance for substrate transport into the active site through electrostatic interactions with the carboxylic groups of hyaluronan. Three amino acid residues (Asp107, Phe108, Glu109, according to the Pp-Hyal sequence) are extremely conserved and present in the active sites of all hyaluronidases [9]. Only the 3D-structure from *A. mellifera* hyaluronidase (Api m 2) was solved with the substrate hyaluronan, enabling the identification of the active site and points of contact with the substrate [9]. In Api m 2, the residues Asp111 and Glu113 are highly conserved in the substrate-binding site and are proton donors essential for the catalysis [64]. The structure of the complex enzyme-substrate suggests an acid–base catalytic mechanism, in which Glu113 is the proton donor and the N-acetyl group of hyaluronan acts as the nucleophile [64].

The residues Asp111, Tyr184, Trp301 are essential for the positioning of the substrate’s carbonyl of the acetamido group [21]. Tyr227 is responsible for the specificity for hyaluronan and Cys227 substitution is responsible for the chondroitinase function [21].

### Arachnida venom hyaluronidases

#### Scorpion venom hyaluronidases

Scorpion venom hyaluronidases were first identified in 1975 in the venom of the South Indian scorpion *Heterometrus scaber* [69]. Although several studies have demonstrated the presence of hyaluronidases in scorpion venoms, few studies have reported their isolation from these sources [70–72]. This may happen because hyaluronidases are difficult to isolate, only small amounts of them are found in venoms (when compared to other toxins) and their enzymatic activity is abolished very easily [73]. These enzymes were isolated for the first time in 1990 from the venom of *H. fulvipes* in two chromatographic steps: molecular exclusion and cation-exchange chromatography [53]. Six hyaluronidases were isolated from *H. fulvipes* [53], *T. serrulatus* [10, 62], *Palamneus gravimanus* [11], *T. stigmurus* [74] and *M. martensii* [75] venoms and had their biochemical and structural characterization performed.

Currently, the application of “omics” techniques has enabled the identification of new compounds present in animal venoms. There are 12 and 10 known primary sequences deposited in the NCBI and UniProt databanks, respectively, for scorpion hyaluronidases (Table 1). Only two of them correspond to complete sequences: one from *T. serrulatus* venom [Swiss-Prot: W0HFN9] and the other from *M. martensii* venom [Swiss-Prot: P86100] [62, 76]. These protein sequences were deduced from cDNA sequences.

The molecular mass of scorpion venom hyaluronidases may range from 45 to 82 kDa [10, 53, 62]. Generally, they show maximum activity in pH between 4 and 6 and temperatures from 30 to 37 °C. Considerable loss of the hyaluronidase activity is observed at temperatures above 40 °C [10, 11, 53, 75]. The hyaluronidase activity can also be inhibited by heparin, as reported for the enzyme from the scorpions *H. fulvipes*, *P. gravimanus* and *M. martensii* [11, 53, 75]. Furthermore, dithiothreitol (DTT), some ions such as Cu^2+^ and Fe^3+^, and flavonoids are also able to inhibit the hyaluronidase activity [10, 53, 75]. Interestingly, the activity of these enzymes may vary among different species and changes may occur in a diet-dependent manner [77, 78]. However, distinct geographical areas had no influence on the enzyme activity [79].

#### Spider venom hyaluronidases

The first spider hyaluronidases, that are similar to the testicular enzyme, were reported in the venoms of the Brazilian species *Lycosa raptoral* and *Phoneutria nigriventer* in 1953 [80]. However, the first spider venom hyaluronidase was only isolated in 1973 from the tarantula *Dugesiella hentzi* (Girard) and was reported as the major constituent of this venom [34]. Other spider venom hyaluronidases were isolated from *Loxosceles reclusa* [81], *Hippasa partita* [16], *Bracchypelma vagans* [82] and *Vitaluis dubius* [83]. Additionally, the hyaluronidase activity was detected in several other spider venoms [84–89]. Moreover, three spider venom hyaluronidases from *L. leata* [90], *Bracchypelma vagans* [82] and *L. intermedia* [55] were expressed in heterologous systems.

There are four and three known primary sequences deposited in the NCBI and UniProt databanks, respectively, for spider hyaluronidases (Table 1). The complete sequence of the enzyme from *L. intermedia* [Swiss-Prot: R4J7Z9] was obtained from its venom gland transcriptome [55]. The enzyme from *P. keyserlingi* [Swiss-Prot: P86274] had the first 32 amino acid residues from its N-terminal identified by Edman degradation [91].

Spider venom hyaluronidases present a molecular mass that ranges from 33 to 47 kDa in their monomeric form [16, 34, 55, 81–83] and maximum enzymatic activity at 37 °C in pH from 4 to 6 [16, 34, 83, 92]. Spider venom hyaluronidases also show high specificity to hyaluronan, weak activity upon chondroitin sulfate A and an almost absence of activity upon chondroitin sulfates B and C [55, 82, 83]. The activity of these hyaluronidases is inhibited by metal ions, such as Fe^3+^ and Cu^2+^, divalent cations, temperatures above 60 °C and extreme levels of pH (under 4 and over 8) [16, 81, 83]. The processes of thawing and freezing do not seem to influence the stability of the enzyme from *D. hentzi* and *H. partita*, whereas the enzyme from *V. dubius* venom had its activity decreased after a series of thawing and lyophilization cycles [16, 34, 83, 89].

### Chilopoda venom hyaluronidases

Centipedes contain a venom gland connected to a pair of forcipules which are used to capture preys. Centipede bites usually cause burning pain, paresthesia, edema and lead to superficial necrosis in human victims [93]. The hyaluronidase activity has also been detected in the scolopendrid centipede venoms [94]. The venoms from *Otostigmus pradoi* and *Scolopendra viridicornis* showed hyaluronidase-active bands of 40–66 kDa and an additional band of 32 kDa was detected in the first venom [93, 94]. There are two complete primary sequences deposited to the *Strigamia* genus in the Uniprot databank (Table 1) although no paper has been published yet.

### Insecta venom hyaluronidases

#### Caterpillar venom hyaluronidases

The larvae of butterflies and moths are called caterpillars. They produce venom in order to protect themselves against predators that are envenomed upon touching them. The composition of the venom is not well known and it varies among different species of caterpillars [95]. The presence of hyaluronidases has been reported in the venoms of *Lonomia obliqua*, *Premolis semirufa* and *Megalopyge urens* [18, 19, 60]. The hyaluronidase activity of the *P. semirufa* venom was measured in the presence of hyaluronan [60]. A hyaluronidase was suggested as the factor behind the Pararama associated phalangeal periarthritis, a serious public health problem among the Brazilian tappers (rubber plantation workers). It is a disease associated with joint immobilization, loss of the cartilage and bone structure and is known to be caused by the *P. semirufa* envenoming [60].

Additionally, lonoglyases are two hyaluronidases found in the *L. obliqua* venom that present 49 and 53 kDa [19]. These enzymes are endo-β*-N*-acetyl-D-hexosaminidases able to degrade hyaluronan and chondroitin sulfate. Lonoglyases show optimal activity from pH 6 to 7 and no activity was detected below pH 5 and over pH 8. Gouveia et al. [19] suggest that the ability of cleaving hyaluronan and chondroitin sulfate linked to the extracellular matrix could explain the effects of the venom, changing the cell adhesion and migration events. Some researchers have speculated that the degradation of the extracellular matrix results from the synergistic effect with other *L. obliqua* venom toxins, leading to local hemorrhage and renal failure [19].

####  Diptera venom hyaluronidases

Hyaluronidase is related to the hematophagic habit of telmophage insects, being found in the saliva of species of the genera *Phlebotomus* and *Lutzomyia* (Table 1). This enzyme extends the feeding lesion and diffuses anti-hemostatic agents into the host tissue, resulting in a microhemorrhage caused by the bite and facilitating the acquisition of blood by the insect [61]. The salivary hyaluronidase may facilitate the spreading of vector-borne microorganisms transmitted by blackflies (Simuliidae), biting midges (Ceratopogonidae) and horse flies (Tabanidae) [61].

####  Hymenoptera venom hyaluronidases

Proteins from social Hymenoptera (bees, wasps, and ants) venoms can trigger serious allergenic reactions in humans, such as pain, itching, inflammation and irritation, which in some cases may lead to death [96]. The hyaluronidase is among the best-studied components from the *Apis* genus [96]. The apian hyaluronidase is a basic glycoprotein (pI 9.0) of 41 kDa rich in aspartic and glutamic acids, containing 7.24 % carbohydrate [12].

Pp-Hyal (*P. paulista* hyaluronidase) is a glycosyl hydrolase comprised of 338 amino acids and shares high sequence identity (80 to 90 %) with wasp venom hyaluronidases of the Northern hemisphere. The mature enzyme presents a theoretical pI of 8.77 and mass of 43,277 Da determined by mass spectrometry analysis [9]. Four isoforms of hyaluronidase were identified in the *P. paulista* venom by two-dimensional SDS-PAGE followed by mass spectrometry [97]. A 3D structural model of the most abundant isoform (Hyal III) was constructed. This isoform contains 288 amino acid residues, 44,340 Da and pI of 9.5 [98]. The comparison between the Hyal III and Pp-Hyal also showed differences in 27 amino acid residues, in the number of disulfide bonds and in the tertiary structure [9]. The levels of hyaluronidase activity in Hymenoptera venoms vary in response to physiological and environmental factors and the presence of isoforms may be an important strategy to mislead the immune system [9]. The absence of carbohydrate moieties in the bee recombinant hyaluronidase polypeptide chain did not change its antibody binding. On the other hand, this structural difference causes protein aggregation due to the partial destabilization of the molecule [99]. A heterogeneous pattern of N-glycosylation of the hyaluronidase Ves v 2 from *V. vulgaris* was shown by mass spectrometry, disclosing peptides with three different patterns of glycosylation sites: one with glycosylation in the positions Asn79 and Asn127; another in the positions Asn79 and Asn99 and the third one with only one glycosylation site in the position Asn99. Because of this variation, the *in vitro* diagnosis of allergic individuals to wasp venom is quite complex [100].

### Heterologous arthropod venom hyaluronidases

Hyaluronidases from different organisms have been expressed in various expression systems such as bacteria, yeast, plants, insects and mammalian cells [28, 55, 65, 82, 101–108].

The first recombinant hyaluronidase ever produced was the Dol m 2, one of the major allergens from the white face hornet *Dolichovespula maculata* [13]. The recombinant Dol m 2 compared to a native hyaluronidase from the bee venom showed a common T cell epitope, which may be one of the reasons why some patients have sensitivity after bee and hornet envenoming [13]. The bee venom enzyme is the most well-characterized hyaluronidase from venoms. It was expressed in 1998 by Soldatova et al. [109] in insect cells, making possible the determination of the first venom hyaluronidase crystal and the characterization of N-glycans by mass spectrometry [64, 110].

### Potential medical and biotechnological applications of arthropod venom hyaluronidases

There are some reports on the medical applications and off-label use of hyaluronidase in several medical fields [32, 40]. Additionally, some hyaluronidases have been studied to enhance the therapeutic index and the local diffusion of anticancer drugs into tissues and tumors [38, 75, 111–117]. Among the arthropod venom hyaluronidases, BmHYA1 (a hyaluronidase isolated from *Buthus martensi* scorpion venom) reduced the expression of CD44 variant 6 in the breast cancer cell line MDA-MB-231 [75].

Furthermore, a hyaluronidase from bee venom was complexed with IgG antibody, which allows the hyaluronidase’s epitope to be recognizable by the antibody and may contribute to the development of novel proteins with reduced immunogenicity to be used as a safer allergen-specific immunotherapy [118]. Recombinant allergens have been used for diagnostic and therapeutic purposes since they are obtained with consistent quality and unlimited amount [119]. Besides that, they can be modified to reduce their allergenicity and to promote beneficial immunologic properties with the aim of reducing IgE-mediated side effects after immunotherapy [119–121]. Distinct allergens which are absent or underrepresented in therapeutic venom preparations may play a key role for the success of immunotherapy [122]. The immunoglobulin E (IgE), present in the serum of allergic patients to the *Polybia paulista* wasp venom, can recognize the recombinant hyaluronidase from *P. paulista* (Pp-Hyal-rec) expressed in *E. coli* system [123]. A heterologous glycosylated hyaluronidase, rVes v 2 from *Vespula* species, expressed in insect cells system, was used to identify wasp venom allergic patients. The specific diagnosis of allergic patients was improved using the basophil activation test (BAT) with the allergen rVes v 2 when compared to the respective specific IgE detection *in vitro* [124]. Moreover, the carbohydrate epitopes present in the glycosylated insect cell-expressed Api m 2 are responsible for antigenic cross-reactivity to bee and wasp venoms [104, 125]. On the other hand, the nonglycosylated *E. coli*-expressed Api m 2 enabled the serologic discrimination of bee and wasp allergy, allowing the correct prescription of venom immunotherapy [125]. These reports demonstrate that recombinant antigens, such as hyaluronidases, have a great immunogenic potential in allergy diagnosis and immunotherapy [123]. In the future, molecules consisting of allergen-derived peptides bound to a viral carrier might be used for prophylactic and therapeutic allergy vaccination, since they are promising vaccines free of IgE- and T cell-mediated side effects [126].

The intranasal administration of hyaluronidase (bovine or isolated from *T. serrulatus* venom) stopped bleomycin-induced lung injury and fibrosis, and decreased the TGF-β production and collagen deposition, which makes hyaluronidase a promising tool for the recruitment of autologous MSC-like cells to the lungs in the treatment of pulmonary fibrosis [127]. This effect could be improved with the use of a delivery system of poly (D,L-lactide-co-glycolide) (PLGA) microparticles (MPs) loaded with hyaluronidase (HYAL-MP) [128].

Finally, inhibitors of the hyaluronidase activity may be used as potential first aid agents in antivenom therapies since the enzyme has a relevant role in systemic envenoming [62].

## Conclusions

Hyaluronidases are a frequent component from Arthropod venoms. They hydrolyze hyaluronan from the extracellular matrix, facilitating toxin diffusion into the tissues of the prey/victims. Although they are not toxins, they indirectly potentiate the toxicity of venoms. Arthropod venom hyaluronidases are potential adjuvants of anticancer drugs and promising tools for the recruitment of autologous MSC-like cells to the lungs in the treatment of pulmonary fibrosis and for the development of novel proteins to be used in allergy diagnosis and immunotherapy. The isolation and characterization of novel arthropod venom hyaluronidases can unravel much more about the role of these enzymes, which justifies the increasing interest on them and on the development of new hyaluronidase-containing drugs and biopharmaceutical products. Moreover, these studies can contribute to the development of more effective antivenom therapies.

## Abbreviations

3D: Three-dimensional
CHO: Chinese hamster ovary
ECM: Extracellular matrix
GlcNAc: *N*-acetyl-β-D-glucosamine
GlcUA: β-D-glucuronic acid
HYAL-MP: Microparticles loaded with hyaluronidase
MPs: Microparticles
PDB: Protein data bank
PLGA: Poly (D,L-lactide-co-glycolide)
rHuPH20: Recombinant human PH-20 hyaluronidase
